# Case report: Duplicated appendicitis with history of cloacal exstrophy causing bowel obstruction^☆^

**DOI:** 10.1016/j.ijscr.2023.108437

**Published:** 2023-06-23

**Authors:** Jonathan D. Lee, Marla A. Sacks, Andrei Radulescu, Donald Moores

**Affiliations:** a Loma Linda University Health, Loma Linda, CA, United States of America; b Division of Pediatric Surgery, Loma Linda University Children's Hospital, Loma Linda, CA, United States of America

**Keywords:** Duplicated appendix, Bladder/cloacal exstrophy, Appendicitis, Prophylactic appendectomy, Case report

## Abstract

**Introduction:**

Cloacal and bladder exstrophy are rare embryological defects that can cause developmental disruption of surrounding organ structures, the pelvis, spinal cord, and small intestines being the most commonly affected. Duplicated appendix is another rare embryological defect that has historically caused confusing clinical presentations. Our case highlights a rare instance of a patient with cloacal exstrophy who presented with a bowel obstruction and an associated inflamed duplicated appendix.

**Case presentation:**

A newborn male is born with omphalocele-exstrophy-imperforate anus-spinal defects (OEIS) complex. As primary surgical reconstruction was pursued, the patient was found to have a non-inflamed duplicated appendix, which was left unremoved. In the following months, the patient experienced episodes of small bowel obstruction, eventually requiring surgical intervention. During this operation, the duplicated appendix was noted to be inflamed, prompting removal of both appendices.

**Discussion:**

This case highlights the increased prevalence of duplicated appendix in a patient with cloacal exstrophy, as well as the utility of prophylactic appendectomy for patients incidentally found to have a duplicated appendix intraoperatively. The duplicated appendix may lead to increased rates of complications and atypical presentation of appendicitis, supporting the practice of prophylactic appendectomy in patients with an incidentally found duplicated appendix.

**Conclusion:**

We suggest clinicians be aware of the association and potentially atypical presentation of appendicitis in patients with a duplicated appendix, particularly in the setting of cloacal exstrophy. The decision to prophylactically remove an incidentally found, non-inflamed duplicated appendix may be beneficial in preventing confusing clinical presentations and future complications.

## Highlights

• Acute appendicitis commonly effects pediatric patients
• Bladder and cloacal exstrophy are associated with developmental abnormalities, such as a duplicated appendix
• A duplicated appendix may cause confusing clinical presentations and complications such as bowel obstruction
• If a duplicated appendix is incidentally identified, clinicians should consider prophylactic removal of both

## 1 Introduction

Bladder exstrophy is an embryological defect in which the bladder and urethra herniate through a defect in the ventral abdominal wall. The prevalence of bladder exstrophy worldwide is approximately 2 per 100,000 live births, occurring 2–3 times more frequently in males [1]. Although isolated bladder exstrophy may occur, bladder exstrophy is frequently associated with epispadias given the similar embryologic origin. Similarly, cloacal exstrophy is an embryological defect that also presents with urogenital herniation through the abdominal wall. However, being that the cloaca pertains to the undifferentiated common cavity for both intestinal and urinary systems, cloacal exstrophy presents more severely with concomitant intestinal abnormalities versus isolated bladder defects seen in bladder exstrophy. Occasionally, cloacal or bladder exstrophy may present severely with embryological disruption of neighboring structures, commonly disrupting a specific pattern of structures. This pattern of developmental abnormalities is called omphalocele-exstrophy (bladder/cloacal)-imperforate anus-spinal defects (OEIS) complex, which occurs in approximately 1 per 100,000 live births [1].

Duplicated appendix is an anomaly that occurs in approximately 4–9 per 100,000 population vs 1 per 100,000 of OEIS in the normal population [[1], [2], [3]]. Although rare, the wide range of appendiceal variations can lead to atypical presentations of appendicitis as well as increased difficulty in visualization of both appendices on diagnostic imaging [4]. This may lead to delays in diagnosis and surgical management, ultimately promoting the potential for more frequent complications [5]. In this case report, we present a unique instance of an infant male born with OEIS complex without spinal involvement, but with the incidental finding of a duplicated appendix. The following work has been reported in line with the SCARE criteria [6].

## 2 Case report

**Fig. 1:**
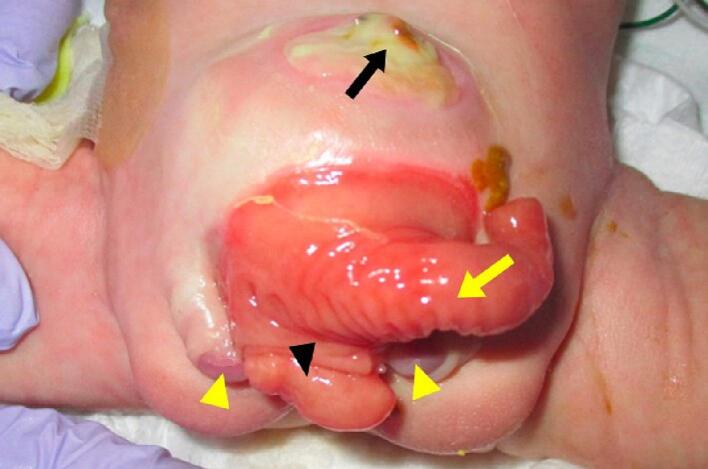
Day of life 1 — omphalocele (black arrow), prolapsed terminal ileum (yellow arrow), hemibladder (black arrowhead), bifid glans (yellow arrowheads). (For interpretation of the references to color in this figure legend, the reader is referred to the web version of this article.)

This is the case of a male infant born prematurely at 36-weeks and 6-days estimated gestational age to a 27-year-old mother G1P0 with a history of polycystic ovary syndrome, and a low-grade cervical lesion associated with human papilloma virus co-infection. The mother smoked cigarettes and used marijuana continuously during the pregnancy. Prenatal care with folate, iron, and routine ultrasounds was begun during the first trimester. At 35 weeks, an ultrasound showed symmetric intrauterine growth restriction and an abdominal wall defect. Labor began following premature rupture of membranes and was associated with late decelerations on fetal monitoring, prompting delivery by caesarean-section. At birth, the patient weighed 2070 g with a head circumference of 31 cm (<10th percentile). Physical exam showed ambiguous genitalia, bladder exstrophy, imperforate anus, and an omphalocele (Fig. 1).

External evidence of myelomeningocele was not present and further spinal imaging was unremarkable. Operative repair was performed at eight months of age, once an adequate goal weight of 3500 g (∼7 lbs 11 oz) had been achieved, at which point the patient underwent modern staged repair for bladder exstrophy (MSRE) and ostomy, as described by Inouye et al. [7]

The major steps of the procedure included takedown of the cecal plate, tubularization of the cecum, and colostomy formation. During manipulation, the patient was noted to have a duplicated bifid cecum with a non-inflamed duplicated appendix, which was not removed. Additional intraoperative findings included the presence of complete duplicated bladders, each with a bladder neck and associated urethral plate connecting with a bifid phallus and hemi glans. The postoperative course was uneventful, and the patient was discharged.

**Fig. 2:**
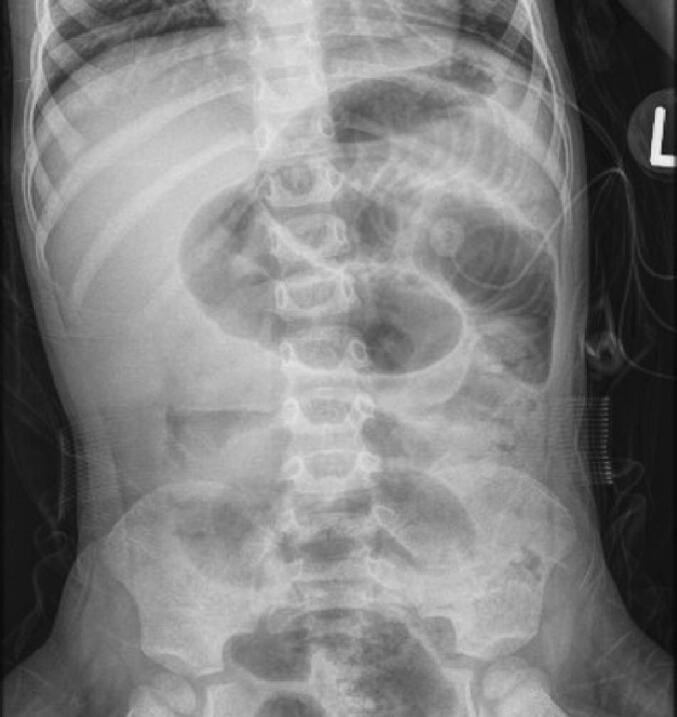
Abdominal radiograph showing dilated loops of bowel concerning for obstruction.

When the patient was two-years and eight-months-old, he experienced several episodes of non-bloody, non-bilious emesis after feeding and was hospitalized with evidence of a bowel obstruction as seen on abdominal radiograph (Fig. 2).

**Fig. 3:**
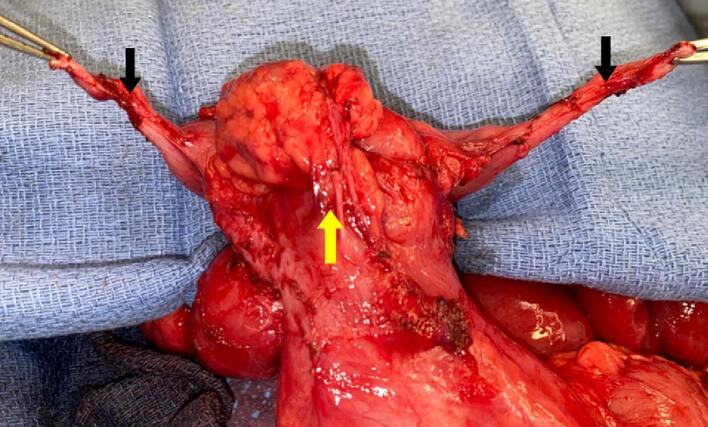
Duplicated appendix (black arrows) attached to the cecum (yellow arrow). (For interpretation of the references to color in this figure legend, the reader is referred to the web version of this article.)

A nasogastric tube was placed and arrangements were made to proceed with operative intervention. The patient was significantly distended so open exploration was pursued over laparoscopy. Exploration revealed diffuse adhesions, particularly in the pelvis, and in internal hernia through a mesenteric defect with an obstructed loop of bowel, as well as significant malrotation. The bowel in the internal hernia was reduced then extensive lysis of adhesions allowed mobilization of the entire bowel from the duodenum to the colostomy. This included exposure of the cecum where duplicated appendices and numerous enlarge mesenteric lymph nodes were identified (Fig. 3). One of the appendices was actively inflamed. Both the appendices were removed, along with a few of the adjacent enlarged lymph nodes, then the abdomen was closed primarily.

The child's post-operative course was uncomplicated, with return of bowel function and adequate urine output by post-operative day three. The patient's nasogastric tube was subsequently removed and oral intake was advanced, which the patient tolerated well until he was discharged on post-operative day eight. At a postoperative clinic visit, the child was recovering well without complication and was awaiting the second stage of the exstrophy repair with pelvic osteotomy.

## 3 Discussion

The majority of duplicated appendices are found incidentally during procedures not primarily involving the appendix [[2], [3], [4]]. Historically, patients who were incidentally found to have a non-inflamed duplicated appendix did not undergo appendectomy unless at least one appendix was inflamed, in which case both would usually be removed [3]. In our case, the duplicated appendix was identified during the patient's first procedure but was not removed due to its normal appearance. During the patient's exploratory laparotomy for suspected small bowel obstruction, the appendix appeared inflamed which prompted removal. Our case prompts the question of whether prophylactic appendectomy in patients found intraoperatively to have a non-inflamed duplicated appendix should be performed to prevent future complications.

Because of the multiple anatomical variations of duplicated appendices, their potential for misdiagnoses and atypical clinical presentation when inflamed is high [8]. Identification of the anomaly can be further confounded when the clinical presentation mimics other conditions, such as adenocarcinoma of the colon, small bowel obstruction, volvulus, or intussusception [9,10].

Regardless of anatomical orientation, the rate of appendiceal inflammation is roughly equal between either of the duplicated appendices. Anteriorly oriented appendices are easily identified, however, patients who experience inflammation of a more poorly visualized appendix (commonly retrocecal), have potential for their appendicitis to be missed intraoperatively due to the presence of a normal appearing anteriorly oriented appendix [4,11]. This can lead to confusing clinical scenarios where a patient with a history of an appendectomy presents with signs and symptoms of recurrent appendicitis [4,5]. Even when the duplicated appendix is identified, removal of the inflamed appendices has increased risk for complications such as stump appendicitis, a postoperative complication with a reported incidence of 1 in 50,000 cases [12]. Previous literature has listed a duplicated appendix as being one of the predisposing anatomical risk factors for stump appendicitis, likely due to the larger surface area of the appendiceal base that is required to be resected [12]. For these reasons, duplicated appendices may carry challenges in management during periods of acute inflammation.

In the case of patients with cloacal exstrophy, patients present frequently with duplicated or bifid organs [13]. Although the direct pathogenesis is unknown, one of the most widely accepted theories explaining cloacal exstrophy proposes that during formation of the lower abdominal wall, the cloacal membrane may overdevelop, disrupting the migration of supportive mesenchymal cells to the midline abdominal tissue [13]. This promotes weakness in the region overlying the bladder wall, allowing herniation of the urogenital sinus through the cloacal membrane during early weeks of embryologic development. This herniation causes abnormal development of the surrounding structures commonly seen in OEIS complex [13,14]. Physicians caring for patients with a history of bladder or cloacal exstrophy should be aware of the possibility of a duplicated appendix, especially ones presenting with abdominal pain and signs of infection.

Taking these potential risks and future intraoperative confusion into consideration, if a patient is already undergoing scheduled abdominal surgery and is found incidentally to have a duplicated appendix, it may be worth removing both appendices, even if they are not inflamed [15]. This point is emphasized by an interesting case report published by Azzam et al. where two infants presenting with bilious emesis are found to have duplicated jejunal cysts with concomitant midgut volvulus [16]. During Ladd's procedure for the malrotation, prophylactic appendectomy was also completed. This report highlights how patients with duplicated enteric organs may be at increased for obstructive pathology, thus when given the opportunity, prophylactic appendectomy to remove a potential foci for recurrent bowel obstruction is recommended. Being that appendicitis is a common condition, our case aims to highlight this less common anatomical abnormality, particularly in patients with cloacal exstrophy and complicated abdominal anatomy, providing possible support for the practice of prophylactic appendectomy in patients with duplicated appendices.

## 4 Conclusion

Duplicated appendix is an infrequent anatomical variant that is occasionally associated with other organ duplications and malformations. Children with congenital anomalies such as cloacal exstrophy deserve a thorough assessment for additional anatomical variations, and if exploration reveals a duplicated appendix, removal of both appendices at that time is recommended. While removal of an inflamed appendix is clearly indicated, prophylactic removal of a non-inflamed duplicated appendix may prevent confusing clinical presentations and potential complications. If a duplicated appendix is found incidentally during initial procedure, removal of both appendices is recommended at that time, regardless if they are inflamed or not. The decision to prophylactically remove an incidentally found non-inflamed duplicated appendix may be beneficial in preventing confusing clinical presentations and complications in the future.

## Funding

No sources of funding.

## Ethical approval

IRB# 5230211 – exempt from IRB approval or review.

Institutional Review Board – human research & compliance.

Loma Linda University Health 24887 Taylor Street Suite 201, Loma Linda, CA 92350.

Reason: not a systematic investigation, not designed to develop or contribute to generalizable knowledge.

## Consent

Written informed consent was obtained from the patient for publication of this case report and accompanying images. A copy of the written consent is available for review by the Editor-in-Chief of this journal on request.

## CRediT authorship contribution statement

Jonathan Lee – data collection, writing paper, data analysis, images.

Marla Sacks – Study design, data collection, edits.

Andrei Radulescu – Study concept, study design, editing paper, provided care for patient.

Donald Moores – Study concept, study design, editing paper, primary surgeon in operation.

Dr. Daniel Moore – Guarantor.

Dr. Andrei Radulescu – Guarantor.

## Declaration of competing interest

No conflict of interest.
